# The Future Extreme Temperature under RCP8.5 Reduces the Yields of Major Crops in Northern Peninsular of Southeast Asia

**DOI:** 10.1155/2022/1410849

**Published:** 2022-03-30

**Authors:** Teerachai Amnuaylojaroen, Nichapa Parasin

**Affiliations:** ^1^Department of Environmental Science, School of Energy and Environment, University of Phayao, Phayao 56000, Thailand; ^2^Atmospheric Pollution and Climate Change Research Unit, School of Energy and Environment, University of Phayao, Phayao 56000, Thailand; ^3^School of Allied Health Science, University of Phayao, Phayao 56000, Thailand

## Abstract

This study explores the impact of rising near-future temperatures on crop yields, particularly rice and maize, in northern Thailand between 2020 and 2029. The potential for high temperatures in northern Thailand between 2020 and 2029 under the Representative Concentration Pathways 8.5 (RCP8.5) scenario indicates that Thailand experienced hot trends between 2020 and 2029 as measured by the annual maximum value of daily maximum temp (TXx), annual minimum value of daily maximum temp (TXn), annual minimum value of daily minimum temp (TNn), and annual maximum value of daily minimum temp (TNx). Northern Thailand had the most dramatic changes in TXn and TNn. Furthermore, TXn levels were found to be significantly higher in northern Thailand. The number of days when TX < 10th percentile (TX10p) intensity decreased, while the number of days with TN < 10th percentile (TN10p) intensity was increasing. The number of days when TN > 90th percentile (TX90p) has become increasingly rare in northern Thailand. The TN90p was dropping in northern Thailand, whereas the Warm Spell Duration Index (WSDI) was growing. Additionally, the cold spell duration index (CSDI) continues to decline. This indicates that the heat persistence index is increasing in northern Thailand. Temperature rises are the most likely to have a detrimental impact on agricultural production, and climate models can predict regional temperature changes with more precision than precipitation. Throughout the planting season (June-December), average yearly temperatures in rice and maize growing areas have climbed by 0.5–0.6°C. The impact estimates for maize and rice are generally negative, that is, −10 ± 4.6% per °C and −8 ± 3.5% per °C, respectively.

## 1. Introduction

Prediction of global warming appeared in the 1980s as an unprecedented high priority that could endanger future human societies [1–3]. The Earth atmosphere's General Circulation Models (GCMs) anticipated that when the fast-increasing CO_2_ concentration in the atmosphere doubled from the beginning of the past century, the global mean temperature would rise by more than 4°C. Many international studies for impact assessments were undertaken between the 1980s and the early 1990s [4–9]. According to the Intergovernmental Panel on Climate Change's Sixth Assessment Report, the new estimates of the chances of crossing the global warming level of 1.5°C in the next decades limit warming to close to 1.5°C, thus even 2°C will be beyond the reach of most. Extreme events from climate change negatively impact agricultural yield and total factor productivity [10, 11]. In recent years, there has been a dramatic increase in research to evaluate extreme weather in agriculture [12, 13]. The increasing frequency or intensity of extreme weather events may increase the risk of multiple simultaneous crop failures within regions or globally [14, 15]. Quantifying yield loss anomalies at large spatial scales and understanding their climatic drivers are prerequisites to assessing vulnerabilities and designing adaptation measures to increase the resilience of food systems [16]. Droughts and heat waves, for example, can have a detrimental effect on agricultural production and have ramifications for communities' livelihoods and food security. Not only countries directly affected by the extreme event are impacted, but also regions throughout the world, which may experience indirect impacts such as lower agricultural exports and increased food costs [17, 18].

Crops are sensitive to climate change, including changes in temperature and precipitation, and to rising atmospheric CO_2_ concentrations [19, 20]. Among the changes, temperature increases have the most likely negative impact on crop yields [21, 22], and regional temperature changes can be projected from climate models with more certainty than precipitation. Agricultural production is vulnerable to climate change. Understanding climate change, especially the temperature impacts, is critical if policymakers, agriculturalists, and crop breeders are to ensure global food security. Especially, rice is Asia's most important crop, supplying a significant amount of dietary energy and employment for the region's 40 billion people. Rice production should be increased further to fulfill the rising demand induced by population growth and economic development. However, the increase in rice production stalled around 1990, when the spread of “Green Revolution” technologies that contributed to the increase in yield was practically halted. While breaking through rice production stagnation was a severe problem for scientists, global warming projections brought another difficult condition to Asian rice cultivation. The main concern is predicting the effects of projected global warming on Asian rice production and developing adaptive rice-producing technology [23, 24].

The expected global warming caused by a rise in greenhouse gas concentrations in the atmosphere is another huge element that makes future food security in Asia questionable [4, 5, 6]. Human activities such as fossil fuel combustion, industrial production, and land-use changes have resulted in rapid increases in the concentrations of carbon dioxide, methane, and nitrous oxide in the atmosphere. According to the report by Intergovernmental Panel on Climate Change (IPCC), atmospheric greenhouse gas levels have climbed to levels not seen in at least 800,000 years, and CO_2_ has increased by 40% since preindustrial times [25]. Many General Circulation Models (GCMs) of various topologies have been built since the original studies on GCMs that predicted global warming induced by an increase in carbon dioxide. The IPCC's fifth assessment report [25] depicted the global climate changes expected by multiple GCMs under four Representative Concentration Pathways (RCPs) of greenhouse gases. The four RCPs used radiative forcing of 2.6, 4.5, 6.0, and 8.5 W/m^2^ of greenhouse gases by the year 2100, corresponding to atmospheric CO_2_ of 421, 538, 670, and 936 ppm, respectively. The expected temperature increase is heavily dependent not only on RCP scenarios, which reflect future human efforts to reduce anthropogenic CO_2_ emissions but also on GCMs. When evaluated at the same level of carbon dioxide, the ranges of the increase in annual mean surface air temperature in the year 2100 predicted by several GCMs are 0.3–1.7°C for RCP 2.6, 1.1–2.6°C for RCP 4.5, 1.4–3.1°C for RCP 6.0, and 2.6–4.8°C for RCP 8.5 [25].

According to recent reports by reference [26], the temperature change will tend to increase by 0.62°C per decade in the future. This could lead to an increase in the heat index of 2.57°C if the temperature increases by up to 1.5°C in Thailand. For this reason, it's important to identify vulnerable areas and develop adaptation strategies for rice culture in the context of environmental warming, as well as ensuring future food security in the response to rising population and rice demand, despite the high degree of uncertainty surrounding the predicted future climate. Our study, by compiling extensive results from the Regional Climate Model and the Decision Support System for the Transfer of Agrotechnology (DSSAT), shows the impact of higher temperatures on reducing crop production, with important implications for developing crop and region-specific adaptation strategies to ensure the future food supply.

## 2. General Information

To reveal the results of temperatures reducing crop production, we used output from both the simulation of the Nested Regional Climate Model (NRCM) [27] for temperature data and the Decision Support System (DSSAT) [28] for rice and maize production under RCP 8.5. The data used from the model's output were described in Sections 2.2 and 2.3, respectively, for NRCM and DSSAT. RCP 8.5 is consistent with current emissions trends and with the path supported by policymakers, who promote the prolonged use of fossil fuels such as coal-fired power. However, projecting decades ahead, emissions on the present business-as-usual track may eventually approach, but not quite reach, the levels often associated with RCP 8.5 (https://climatenexus.org/climate-change-news/rcp-8-5-business-as-usual-or-a-worst-case-scenario/). The analysis begins with the extreme temperature, then the simulated temperature and rice and maize production were shown the relationship. The simulated temperature (1990–1999, 2020–2029) from the NRCM simulation under RCP 8.5 was used as input data for nine World Meteorological Organization Expert Team on Climate Change Detection and Indices (WMO-ETCCDI) recommended extreme temperature indices, including the annual maximum value of daily maximum and minimum temperatures (TXx and TNx), the annual minimum value of daily maximum and minimum temperatures (TXn and TNn), cool nights (TN10p), warm nights (TN90p), and cool day (CSDI) to analyze temperature extremes as listed in Table 1.

### 2.1. Study Area Description

**Figure 1:**
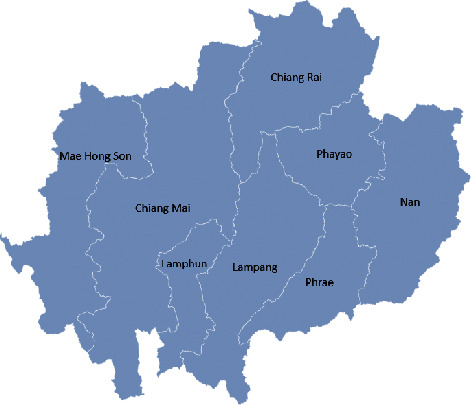
Northern Thailand.

Northern Thailand (Figure 1) is predominantly hilly and is the source of many of the country's rivers, including the Chao Phraya, which is formed by the confluence of four rivers: the Ping, Wang, Yom, and Nan. Summer storms are frequently encountered as a result of the natural characteristics of high mountains, steep river basins, and highlands. The northern mountains are cut into the center plain by steep river valleys and peaks. These inherent qualities have historically facilitated a variety of agricultural practices, including rice farming in valleys and upland moving cultivation. This area is influenced by southwest and northeast monsoons. It was demonstrated by separating into 2 seasons (wet season: May to October; dry season: November, December, January to April) [29]. Rice is a vital food crop in the upper northern provinces. To be precise, the grade of Jasmine rice (Khao Dawk Mali (KDML) 105) is worldwide known, with an annual rice yield area of around 405,801 hectares and 1,487,506 tons. The seeding season lasts from June to October, and the harvesting season lasts from November to December. Typically, maize is seeded following the rice season.

### 2.2. Information of NRCM Output

We use the NRCM modeling system output [27] in combination with the Price Weller Pinkel (PWP) model [30] to simulate both current-day (1990–1999) and near-future (2020–2029) climatology under the Representative Concentration Pathway (RCP) 8.5 scenario. The CCSM version 4 is used as the meteorological initial and boundary conditions in the model [31]. The NRCM is a regional climate model that is forced with the CCSM and is based on the Weather Research and Forecasting Model (WRF) [31–34]). It is similar to the WRF in that the beginning and boundary conditions are determined using a small area. The PWP model is an oceanic bulk mixing-layer model that combines convection adjustment and mixing layer shear instability. It is presently being used in the Hybrid Coordinate Ocean Model (HYCOM) for vertical mixing [35]. The model was set up in the manner outlined by Amnuaylojaroen et al. [36]. The Runge–Kutta integration approach [32] incorporates several meteorological parameters, including wind, temperature, water vapor, and cloud hydrometeors. Furthermore, the model calculated the feedback and evolution of atmospheric aerosols on both short- and long-wave radiation using the Rapid Radiative Transfer Model (RRTMG) radiation schemes [37]. Simultaneously, the model incorporates aerosol feedback effects on meteorology processes, such as the impacts of aerosols on clouds and precipitation, which are estimated using the Thompson method [38]. In the simulation, subgrid-scale convection was handled *via* the Grell-3 method. The Noah Land Surface Model was used to calculate the land-atmosphere interactions [39]. Grid nudging [40] was used in the outer domain for all vertical levels in the model with nudging coefficients of 0.0003 s^−1^ for all variables including horizontal wind, temperature, and water vapor every 6 h for realistic large-scale meteorology. The domains cover the majority of Southeast Asia as well as parts of China. The outside domain (10°S 25°N, 83–118°E) has a grid spacing of 60 km, while the inner region (5–20.5°N, 96–106°E) has a grid spacing of 10 km. From the surface to 10 hPa near the tropopause, the vertical level was set to 53 levels. For forced beginning and boundary conditions from the outside domain to the inner domain, a one-way nesting mechanism was used. The model was performed for two scenarios: (1) a current simulation (1990–1999) and (2) a future simulation (2020–2029).

### 2.3. Information of DSSAT Output

We used the output of rice and maize data from the Decision Support System (DSSAT) version 4.7.5 simulation from June to December 2020–2029 [28]. DSSAT output was confirmed using on-farm data from Thailand's Department of Agriculture from 2010 to 2018. The seeding season lasts from June to October, and the harvesting season lasts from November to December. The seasonal growth, development, and yield of crops, as well as the change in soil, water, carbon, and nitrogen balance under the farming system, were determined [41]. Rice and maize production in northern Thailand were estimated using the Crop Environmental Resource Synthesis-Rice (CERES-Rice) [42] and Crop Environmental Resource Synthesis-Maize (CERES-Maize) [43] models incorporated in DSSAT. KDML105 rice cultivar and short-season maize cultivar were employed in the study. Buddhaboon et al. [44] and Lana et al. [45] revealed genotype coefficients for the cultivars KDML105 and short-season maize (SW3601). The International Benchmark Sites Network for Agrotechnology Transfer (IBSNAT) standard data collection procedures were followed [46]. The genetic parameters were estimated using the “genotype coefficient calculator” given by the DSSAT shell [47], whereas the genetic coefficient for maize was based on references [48, 49]. The CERES-Maize model was validated at the Phitsanulok Field Crops Experiment Station (PSL FCES) on various planting dates between 1997 and 1999, with fertilizer levels based on the Department of Agriculture of Thailand's recommendations of N: 6 kg/ha, P_2_O_5_: 7.5 kg/ha, and K_2_O: 7.5 kg/ha. The soil analysis layers had a bulk density of 1.035 g/cm^3^, a total organic carbon content of 1.46%, a total nitrogen content of 0.601%, a buffer pH of 5.63, a water pH of 6.59, the extractable phosphorus content of 5.766 mg/kg, the exchangeable potassium content of 5.766 cmol/kg, and a stable organic compound content of 1.46%.

### 2.4. Performance of Model Output

The model achievement was demonstrated statistically in Table 1 using several statistical indicators, that is, the index of agreement (IOA), mean bias (MB), standard deviation of residue (SD), and R-square (*R*^2^). The statistical analysis between model and observed data that included TMD, MERRA, and APHRODITE for temperature and on-farm data for rice and maize production is listed in Table 2. The analysis shows a good performance of the model with R-squared values in ranges of 0.89 to 0.98 for annual data, reflecting the model's high ability to capture temperature trend. Similarly, the IOA values for annual temperature were in the range of 0.79–0.95. The temperature was underestimated in both the dry and rainy seasons as opposed to measurements with MB values ranging from – 0.94 to 0.15°C. The standard deviation of the model's residuals is 1.87–2.11°C for temperature and 226–615 ha for rice and maize production.

## 3. Results and Discussion

### 3.1. Situation of Extreme Temperature in Thailand during 2020–2029

In general, Thailand experienced hot trends between 2020 and 2029, according to the annual TXx, TXn, TNn, and TNx values. The highest changes in TXn and TNn occurred in northern Thailand. TXn levels were also discovered to be significantly higher in northern Thailand. TX10p was on the decline, whilst TN10p was on the rise. TX90P was becoming increasingly scarce in northern Thailand. The TN90P, in particular, was dropping, whilst the WSDI has grown in Thailand's north. CSDI is also declining. This indicates that the heat persistence index was rising in northern Thailand. The frequency of distribution function was used to display the general temperature extreme change. This section quantifies changes in each index, including TXx, TXn, TNn, TNx, TX10P, TN10P, TX90P, TN90P, WSDI, and CSDI.

**Figure 2:**
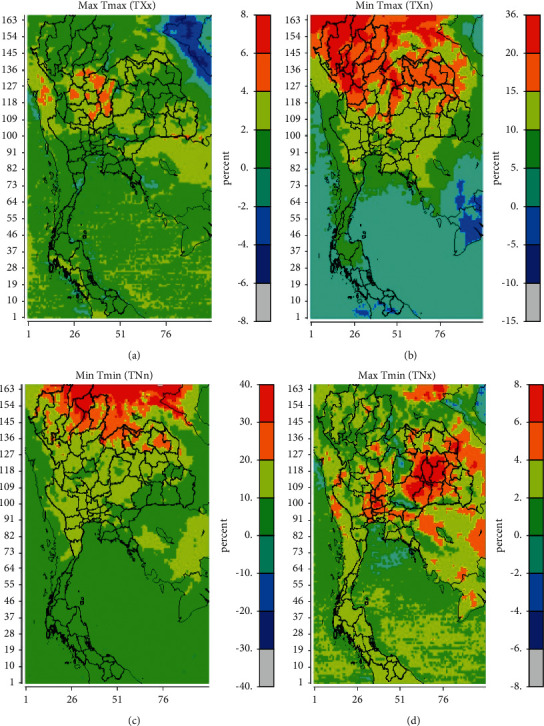
Spatial distribution of percentage differences in (a) TXx, (b) TXn, (c) TNn, and (d) TNx between years 2020 and 2029 and between years 1990 and 1999 in Thailand.

The spatial distribution of the annual TXx, TXn, TNn, and TNx values revealed that there were hot trends in Thailand between 2020 and 2029 (Figure 1). TXx levels generally fluctuate between 2% and 6% per decade, as illustrated in Figure 2(a). Though annual TXn and TNn changes ranged between 10% and >36% per decade (Figures 2(b) and 2(c)), the greatest changes occurred in the north of Thailand, with changes exceeding 36%. TNx discovered the greatest development in the eastern part of Thailand, at 8% per decade (Figure 2(d)). TXx showed a slight upward trend of 6% in the center of Thailand and a downward trend of 2% in the rest of the country. TXn showed a 15% decline in the eastern and central regions of Thailand but a 10% decline in the southern region. Significant statistical increases in TXn levels, ranging from 20% to >36%, were discovered in northern Thailand. TNn showed a downward trend of 20% in the majority of Thailand, except for the tip of northern Thailand, which showed statistically significant increases of >40%. The TNx level increased by 8% in the east of Thailand. This indicates that the minimum maximum temperature values are concentrated in northern Thailand, while the maximum maximum temperature values are marginally increased in Thailand.

**Figure 3:**
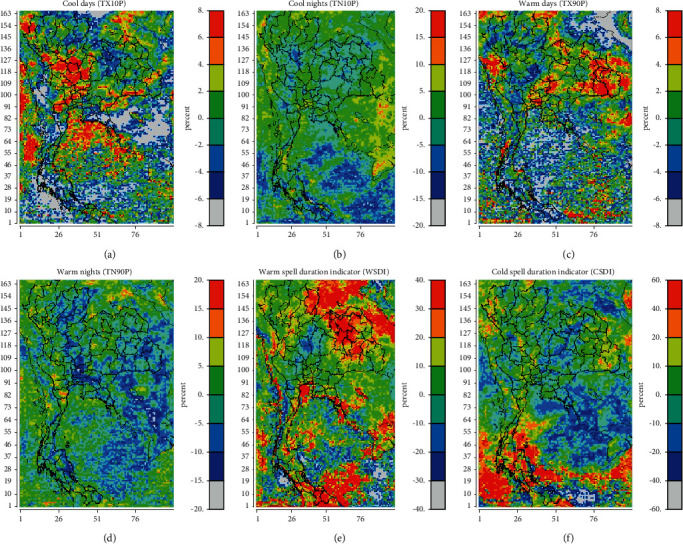
Spatial distribution of percentage differences in (a) TX10P, (b) TN10P, (c) TX90P, (d) TN90P, (e) WSDI, and (f) DSDI between years 2020 and 2029 and between years 1990 and 1999 in Thailand.

**Figure 4:**
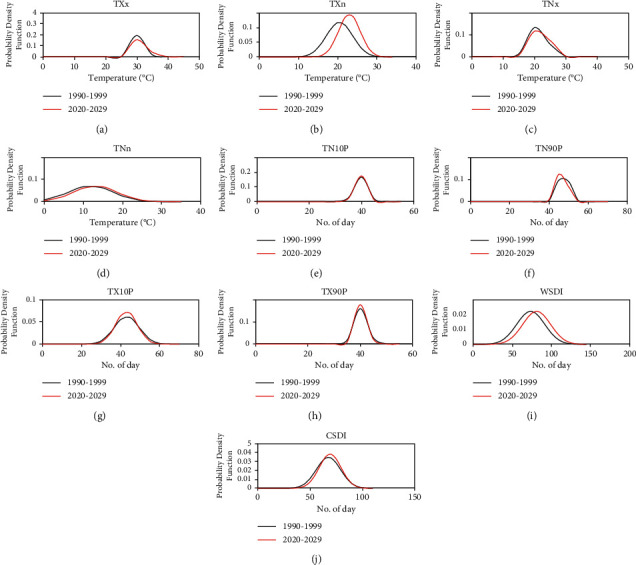
Probability distribution function of the (a) TXx, (b) TXn, (c) TNx, (d) TNn, (e) TN10P, (f) TN90P, (g) TX10P, (h) TX90P, (i) WSDI, and (J) CSDI between the years 2020–2029 and 1990–1999 in northern Thailand.

The spatial distribution of the extreme temperature change pattern in Thailand was depicted in Figure 3 using TX10P, TN10P, TX90P, and TN90P, as well as other indicators such as the WSDI and CSDI. As shown, TX10p is rising at an 8% rate in the center of Thailand and declining at a level of between 2% and 4% in most of Thailand's regions (Figure 3(a)). TN10p demonstrated an upward trend in the majority of Thailand in the range of 5% to 10%. TX90P is rising at a rate of between 2% and 8% in the east of Thailand while decreasing at a rate of between 2% and 4% in the rest of the country (Figure 3(b)). In Thailand, TN90P levels fluctuated between (−5) and 5%. TN90P, in particular, exhibits a 5% decline in some areas of central and northern Thailand (Figure 2(d)). WSDI has increased by more than 40% in some areas of northern and eastern Thailand, while other regions have seen rises of between 10% and 20% (Figure 3(e)). CSDI is decreasing by 10% in the majority of Thailand but significantly increasing at >60% of CSDI in southern Thailand (Figure 3(f)). This demonstrates that while the heat persistence index in northern, central, and eastern Thailand is rising, the cold persistence index in southern Thailand is decreasing.  Figure 4 depicts the frequency distributions of each index for the years 1990–1999 (black line) and 2020–2029 (red line). In general, TXn, WSDI, and CSDI tend to rise between 2020 and 2029, while TN90P tends to fall. TXn tends to increase the temperature in Thailand by 5°C between 2020 and 2029, while WSDI and CSDI tend to increase by about 10 and 5 days, respectively, in the future. Meanwhile, TXx, TNx, TNN, TN10P, and TX90P will change slightly in the future.

### 3.2. Situation of Rice and Maize Production in Northern Thailand during 2020–2029

**Figure 5:**
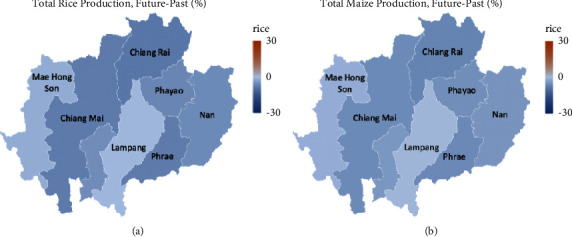
The percentage difference in the years 2020–2029 of total rice and maize production in northern Thailand (adapted from reference [28]).

The future total production of rice and maize during 2020–2029 under the worst climate change scenario (RCP8.5) was shown in Figure 5. In general, there was a decrease in aggregate yields by 5.15% and 3.9% for rice and maize compared to the past simulations. We found that Chiang Rai, Chiang Mai, and Phrae were the most declining yields in the region by about 7%, while Lampang was the only province that had a slight effect on climate change of less than 1%. As reported in reference [27], the future temperature tends to increase about 2–5°C in Thailand. The increased temperatures would have an impact on agricultural irrigation. The result is very statistically significant. As a result of climate change, a decrease in future crop production is likely due to a lack of water for agriculture and the severity of drought conditions. In particular, irrigation is positively associated with improved rice productivity, while temperature increases tend to be sensitive to maize production.

### 3.3. Effect of Temperature Change on Rice and Maize in Northern Thailand During 2020–2029

**Figure 6:**
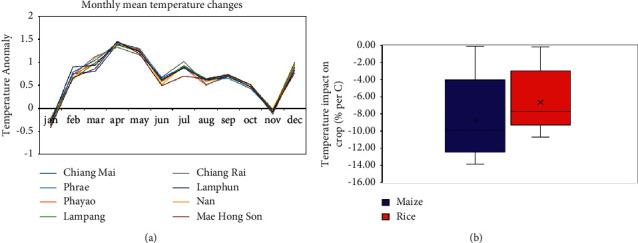
(a) Monthly mean temperature change in 2020–2029 compared to 1990–1999, (b) averaged rice and maize change during planting season in 2020–2029 compared to 1990–1999.

Temperature increases are the most likely to have a negative influence on agricultural yields, and regional temperature changes can be anticipated with greater accuracy from climate models than precipitation.  Figure 6 suggests that mean annual temperatures in rice and maize growing areas have risen by 0.5–0.6°C throughout the planting season (June–December) (Figure 6(a)). As a result, it is vital to assess the impact of rising temperatures on crop production. The temperature-yield relationship was depicted in Figure 3(b) for the eight provinces of northern Thailand. The models predict a detrimental impact for both rice and maize but to various degrees. The impact estimates for maize and rice are generally negative, that is, −10 ± 4.6% per °C and −8 ± 3.5% per °C, respectively. According to the data, a 1°C increase in temperature reduces rice output less than it does maize yield (Figure 6(b)).

### 3.4. Suggestion for Adaptation Strategies

To cope with the effect of higher temperatures on crop production in this region, there were several suggestions from previous studies. For example, the greatest significant advance has been made by studying rice near-isogenic indica and japonica cultivars with early blooming features. Further research is needed to determine the effectiveness of these heat-stress-prone Asian near-isogenic lines in the field. Stable rice production under global warming requires the development of heat-stress-tolerant cultivars. Nevertheless, the early blooming characteristic alone may not be enough to prevent expected heat-induced spikelet sterility under global warming. Studies on heat stress tolerance have also made significant progress, with the mechanism for heat-stress tolerance being clarified (the length of basal anther dehiscence being identified as a genetic feature for tolerance and quantitative trait locus (QTLs) for influencing dehiscence length being identified) [50–56]. More research is needed to generate heat-stress-tolerant genotypes based on the quantitative trait locus (QTLs) found, while also looking for other features that are perhaps linked to heat-stress tolerance. Another point is to take measurements of rice's reactions to high temperatures and carbon dioxide. According to certain research, heat-induced sterility is affected not only by air temperature but also by air humidity due to its transpiration cooling effects on panicles [53, 57]. Under hot and highly dry conditions, spikelet temperatures can be significantly lower than air temperatures, perhaps alleviating heat-induced spikelet sterility. On the other hand, several studies have found that a significant water-vapor pressure deficit in the air has a negative impact on spikelet sterility via desiccation of floral organs. As a result, more study on the direct and indirect impacts of high vapor pressure deficit on rice spikelet sterility during the flowering stage is required to create the foundation for a more general model for forecasting heat-induced spikelet sterility.

### 3.5. Discussion

All indices, especially TXx, TXn, TNx, and TNn, illustrate a clear upward trend, while the TN10P, TN90P, TX10P, TX90P, and CSDI show a clear downward trend (Figure 5). These findings are consistent with Limsakul [58] and also with the warming observed in the regional and global scales [59–62]. Most of the absolute changes in Thailand's averaged extreme temperature indices have a consistent linear relationship with GMT [58]. Temperature fluctuations and extremes on a local scale are almost linearly related to large-scale or global mean temperature changes. Furthermore, Wartenburger et al. [63] found that a number of regionally average climate indices linearly relate to GMT, with the temperature-derived indices and drought and water-cycle indices exhibiting the strongest linear relationships. In general, shifts in indices calculated from daily minimum temperatures are greater than those calculated from daily maximum temperatures, which is consistent with previously recorded regional findings [59–61]. However, the absolute temperature indices (TXx and TNx), except for TNn and TXn, exhibit mixed and weaker patterns. Choi et al. [59] and Caesar et al. [64] noted that these indices display greater interannual variation than other incident indices, owing to their reliance on a single event each year is vulnerable to significant variability. Additionally, northern Thailand experienced a large increase in these absolute temperature indices (TXx, TXn, TNn, and TNx). This finding is consistent with Masud et al. [65] who used the output from a simulation of the PRECIS Regional Climate Model data over northern Thailand. They reported that all temperature extreme indices (TXx, TXn, TNx, and TNn) are increasing significantly.

Increasing the extreme temperature in northern Thailand likely affects agriculture. As discussed in references [27, 66], the north of Thailand may experience severe dry conditions that would likely lead to strong drought events, and it is essentially necessary to plan for irrigation. These favorable conditions tend to affect crop production, especially maize. The results are corresponded to the global trend [67, 68], which suggested that rice productivity under global warming will also decrease as global temperatures increase. The direct negative temperature impact on yield could be additionally affected *via* indirect temperature impacts. For instance, increasing temperature will increase atmospheric water demand, which could lead to additional water stress from increased water pressure deficits, subsequently reducing soil moisture and decreasing yield [56, 69]. However, an accelerated phenology from increased temperatures leads to a shorter growing period and less days of crop water use within a cropping season. Such indirect temperature effects are taken into account in each of the methods but are not explicitly quantified. Other indirect temperature impacts include more frequent heat waves and possible temperature impacts on weeds, pests, and diseases [70, 71]. Increases in management intensity and yield potential could also unintentionally increase yield sensitivity to weather [72]. Higher temperatures have a greater impact on maize than on rice, because temperature increases during maize pollination in hot growing settings threaten to make midway crop loss the norm. In addition to causing a harsher thermal environment, early-season temperature rises have forced the maize reproductive phase to begin earlier, increasing the danger of heat and water stress. Declines in maize maturation time show that, independent of water supply impacts, yield potential is becoming progressively constrained by warming. The timing and magnitude of temperature rises, as well as the features of the growing season, all influence regional variances in effects. The continuation of existing climate trends may result in significant yield reductions in some regions. Farmers could avoid certain losses by changing planting dates and maize varietal types [73].

### 3.6. Limitation of the Study

There were a few shortcomings in this work. This study, for example, has limitations due to the time span studied and the processes addressed. This study examined temperature over a ten-year period, which is insufficient to construct an average climatology and so misses interdecadal variability in temperature. Given the actual restrictions of available resources, this was a necessary constraint and an improvement over previous work that concentrated solely on one-year duration [74, 75]. Furthermore, under a single RCP, one simulation completely ignores the impact of uncertainties and how to handle them. We believe the findings are still useful because they demonstrate how climate change might affect agriculture. Future research should investigate a longer time period, such as 30 years, in order to capture more variability in the atmosphere and conduct several RCPs.

## 4. Conclusion

This study addresses the reduction in crop yield, including rice and maize, resulting from the increase in temperatures in 2020–2029 in northern Thailand using the data from the simulation of NRCM and DSSAT under the worst-case scenario of climate change (RCP 8.5). To evaluate the model's performance before being used to reveal the results of temperatures reducing crop production, the modeled output, including temperature, rice, and maize production, was compared to observations from TMD, MERRA, and APHRODITE for temperature and on-farm data for rice and maize production. The validation of the model results demonstrates remarkable performance, with IOA values for annual temperature ranging from 0.79 to 0.95 and MB values ranging from –0.94 to 0.15°C. The standard deviation of the model's residuals is 1.87–2.11°C for temperature and 226–615 ha for rice and maize production. This study showed that widespread significant trends in extreme temperature indices with clear warming evidence in all indices were found in Thailand between the years 2020 and 2029, especially in northern Thailand where the heat persistence index was increasing, which is more likely to have a detrimental influence on agricultural production. Under the worst climate change scenario (RCP 8.5), future total rice and maize production in northern Thailand decreased in aggregate yields by 5.15% and 3.9%, respectively. Temperature increases are the most likely to have a negative influence on agricultural yields. The increase in temperature has an effect on the reduction of rice and maize production by −8 ± 3.5% per °C and −10 ± 4.6% per °C, respectively, in northern Thailand between the years 2020 and 2029. The adaptation strategies should be applied to improve crop production in northern Thailand in the future [76].

## Figures and Tables

**Table 1:** List of ETCCDMI temperature indices.

Index	Descriptive name	Definitions	Units
TXx	Max Tmax	Annual maximum value of daily maximum temperature	°C
TNx	Max Tmin	Annual maximum value of daily minimum temperature	°C
TXn	Min Tmax	Annual minimum value of daily maximum temperature	°C
TNn	Min Tmin	Annual minimum value of daily minimum temperature	°C
TN10p	Cool nights	Number of days when TN < 10th percentile	days
TN90p	Warm nights	Number of days when TN > 90th percentile	days
TX10p	Cool days	Number of days when TX < 10th percentile	days
1TX90p	Warm days	Number of days when TX > 90th percentile	days
CSDI	Cool days	Cold spell duration index	days
WSDI	Warm days	Warm spell duration index	days

**Table 2:** The statistical analysis comparing the modeled and observed temperatures (adapted from [6, 27, 28]).

Data	Coefficient of determination (*R*^2^)			Mean bias (MB)			Standard deviation of residue (SD)			Index of agreement (IOA)		
	Temperature	Rice	Maize	Temperature	Rice	Maize	Temperature	Rice	Maize	Temperature	Rice	Maize
APHODITE	0.98	—	—	–0.94	—	—	2.02	—	—	0.90	—	—
TMD	0.89	—	—	–0.92	—	—	1.87	—	—	0.76	—	—
MERRA	0.93	—	—	–0.15	—	—	2.11	—	—	0.95	—	—
Observation	—	0.88	0.85	—	−245	−176	—	615	226	—	0.89	0.81

## Data Availability

All model data generated or analyzed during this study are included in this article. The observation data used in this study were provided with permission by the Thai Meteorological Department (TMD) for meteorological data and the Department of Agriculture (DOA) for rice and maize production data and hence cannot be made freely available. Access to these data can be obtained by contacting the TMD at http://www.tmd.go.th/ and DOA at http://www.doa.go.th/.
